# Comparing emergency medical service and walk-in patients in German emergency departments: a prospective multicentre survey

**DOI:** 10.1186/s12873-026-01578-9

**Published:** 2026-04-14

**Authors:** Sarah Oslislo, Kalina Witt, Edgar Steiger, Johannes Hagelskamp, Dominik von Stillfried, Gökhan Katipoglu, Christian Pfeiffer, Michael Dommasch, Matthias Klein, Rajan Somasundaram, Christian Wrede, Michael Bayeff-Filloff, Viktoria Bogner-Flatz, Steffen Herdtle, Markus Zimmermann

**Affiliations:** 1 Central Research Institute of Ambulatory Health Care in Germany (Zi), Salzufer 8, 10587 Berlin, Germany; 2 Bavarian Association of Statutory Health Insurance Physicians (KVB), Elsenheimerstraße 39, 80687 Munich, Germany; 3 Department of Emergency Medicine, Technical University of Munich (TUM), Klinikum rechts der Isar, Ismaninger Straße 22, 81675 Munich, Germany; 4 Emergency Department, Ludwig Maximilian University of Munich, Campus Großhadern, Marchioninistraße 15, 81377 Munich, Germany; 5 Department of Emergency Medicine, Charité – Universitätsmedizin Berlin corporate member of Freie Universität Berlin and Humboldt-Universität zu Berlin, Campus Benjamin Franklin, Hindenburgdamm 30, 12203 Berlin, Germany; 6 Department of Emergency Medicine, Helios Klinikum Berlin-Buch, Schwanebecker Chaussee 50, 13125 Berlin, Germany; 7 MSB Medical School Berlin, Rüdesheimer Straße 50, 14197 Berlin, Germany; 8 Department of Emergency Medicine, RoMed Clinics Rosenheim, Ellmaierstraße 23, 83022 Rosenheim, Germany; 9 Department of Emergency Medicine, Clinical Centre Ebersberg Munich East, Pfarrer-Guggetzer-Straße 3, 85560 Ebersberg, Germany; 10 Department of Acute and Emergency Medicine, Agatharied Hospital, Norbert- Kerkel-Platz, 83734 Hausham, Germany; 11 Emergency Department, University Hospital Regensburg, Franz-Josef-Strauß-Allee 11, 93053 Regensburg, Germany

**Keywords:** Ambulance services, Hospital emergency service, Decision-making, Health services utilisation

## Abstract

**Background:**

Emergency department (ED) crowding is a major concern across Europe. This study compares EMS-transported and walk-in patients across EDs in different settlement types with regard to care-seeking behaviour, presenting complaints and awareness of alternative care pathways.

**Methods:**

We conducted a prospective, multicentre survey among patients in 20 EDs in Berlin and Bavaria (Mar–Oct 2024). A tablet-based questionnaire captured sociodemographics, presenting complaints, perceived urgency and health-care utilisation. EMS and self-referred patients (walk-ins) were compared using χ² and Mann–Whitney U-tests. Multivariable logistic models examined predictors of EMS use and attendance at predominantly urban versus intermediate EDs.

**Results:**

Of 9,719 patients, 2,641 (27.2%) arrived by EMS. EMS patients were older and more likely to attend predominantly urban EDs. They had more often cardio-respiratory or neurological complaints, whereas pain predominated in walk-ins. Prior outpatient contact was less common among EMS patients (29.3%). In adjusted regional analyses, injury-related presentations were more common in predominantly urban EDs (20.5% vs. 26.3%; *p* = 0.003), while cardio-respiratory and abdominal complaints showed no differences. Awareness of national on-call service number (116117) was associated with higher odds of predominantly urban attendance.

**Conclusions:**

EMS patients differ from walk-ins in age, presentation and care-seeking. Differences between regions in case-mix and pre-hospital decision-making suggest that strategies to improve emergency care coordination may benefit from region-sensitive approaches. Whether integration of structured telephone advice (116117) and digital self-assessment tools with EMS dispatch can contribute to more appropriate resource allocation warrants prospective evaluation.

**Registration:**

DRKS00033986; DRKS00034961.

**Supplementary Information:**

The online version contains supplementary material available at 10.1186/s12873-026-01578-9.

## Background

Emergency departments (EDs) across Europe face persistent challenges from rising patient volumes and resource constraints; crowding is linked to delays, adverse outcomes and system inefficiency [1]. Patients transported by emergency medical services (EMS) constitute a substantial share of ED attendances; in Germany, approximately 35% of ED patients arrive by EMS, varying by level of care and region [2]. Germany maintains two national telephone lines for acute and urgent medical care: 112, dedicated to time-critical emergencies (EMS) and 116117, which provides a nationwide, 24-hour patient service for non-life-threatening conditions. Calls to 116117 are subject to structured initial triage, directing patients to the appropriate point of care and enabling scheduling for ambulatory or out-of-hours services. Only in some parts of Germany is this line directly linked to EMS control centres. Moreover, the 116117 service is increasingly used to facilitate teleconsultations with office-based physicians. EMS in Germany follows a physician-based, two-tier model in which paramedic-staffed units are complemented by selectively dispatched on-scene emergency physicians for high-acuity cases. In Germany’s statutory health insurance system, patients have broad freedom of provider choice and may directly self-present to EDs without prior referral; use of the 116117 is recommended but not mandatory. The introduction of some gate-keeping mechanism is on the political agenda, but this is yet not clearly defined.

Patients presenting to the ED via EMS constitute a distinct patient population characterised by differing demographics, clinical presentations and patterns of health-care utilisation compared with self-referred walk-in patients (walk-ins) [3, 4]. Understanding their perspectives – particularly with regard to perceived urgency, motives for ED attendance and receptiveness to alternative care pathways – is essential for optimizing EMS and ED workflows and effective resource allocation. In Germany however, evidence on the perspectives of EMS-transported patients within the ED setting remains limited [3, 5]. This study aims to compare EMS-transported and walk-in ED patients across different settlement types [6] with regard to patient-reported reasons for ED attendance, presenting complaints, prior outpatient contact, awareness of the national medical on-call service (116117) and willingness to engage with digital self-assessment tools.

## Methods

### Study design and setting

We conducted a prospective, multicentre, cross-sectional study in accordance with the STROBE guidelines [7]. Data were collected between March and October 2024 across five EDs in Berlin and 15 in Bavaria, representing different levels of hospital care (from basic and standard care hospitals to tertiary and university medical centres). To ensure recruitment consistency, three smaller EDs in Upper Bavaria and two EDs in Swabia belonging to nearby facilities of the same provider were grouped into two aggregate sites, resulting in 20 participating centres overall (Table 1). The planned sample size of ≈ 10,000 participants (≈ 500 per centre) was designed to allow estimation of a conservative prevalence of 50% with a 95% confidence interval and a standard error of 2%, while accounting for an anticipated 20% drop out rate.

**Table 1:** ED characteristics

ED	Emergency care level	BBSR- Index	Eurostat-Index	Annual patient volume (2024)
Agatharied Hospital	Secondary	rural	intermediate	24,499
Barmherzige Brüder Hospital - Regensburg	Tertiary	urban	intermediate	35,800
University Hospital Regensburg	Tertiary	urban	intermediate	36,800
Hospital Ebersberg Munich-East	Secondary	urban	intermediate	29,445
Garmisch-Partenkirchen Hospital	Secondary	rural	intermediate	30,430
RoMed Hospital - Rosenheim	Tertiary	urban	intermediate	36,321
RoMed Hospital - Prien, Bad Aibling, Wasserburg	Primary	urban	intermediate	46,531
Starnberg Hospital	Secondary	urban	intermediate	23,000
Aschaffenburg Hospital	Tertiary	urban	predominantly urban	46,855
University Hospital Augsburg	Tertiary	urban	predominantly urban	80,605
Fürth Hospital	Tertiary	urban	predominantly urban	53,132
Ludwig-Maximilian University Munich- University Hospital Campus Großhadern	Tertiary	urban	predominantly urban	39,000
Ludwig-Maximilian University Munich- University Hospital Campus Innenstadt	Tertiary	urban	predominantly urban	26,242
Technical University of Munich- University Hospital	Tertiary	urban	predominantly urban	66,976
Wertach Hospital - Schwabmünchen, Bobingen	Primary	urban	predominantly urban	21,956
University Hospital Charité Berlin – Campus Benjamin Franklin	Tertiary	urban	predominantly urban	46,000
Vivantes Hospital Friedrichshain – Berlin	Tertiary	urban	predominantly urban	58,239
Vivantes Hospital Neukölln - Berlin	Tertiary	urban	predominantly urban	65,000
BG Hospital Unfallkrankenhaus Berlin (ukb)	Tertiary	urban	predominantly urban	66,600
Helios Hospital Berlin-Buch	Tertiary	urban	predominantly urban	49,329

BBSR index: German Federal Institute for Research on Building, Urban Affairs and Spatial Development (BBSR) classification of regional settlement structure. Eurostat index: See Methods section for classification details. Abbreviations: ED=emergency department

### Participants and recruitment

All adult patients presenting to the participating EDs during the study period were eligible for inclusion. Patients younger than 18 years were enrolled only if a legal guardian provided consent and completed the questionnaire on the patient’s behalf. Minors were included to capture the full spectrum of ED attendances, as paediatric patients constitute a relevant share of both EMS and walk-in presentations. Exclusion criteria comprised presentation with an immediate, life-threatening condition – defined as Manchester Triage System category “red” or Emergency Severity Index level “1” – as well as inability to provide informed consent (e.g. due to cognitive impairment) or insufficient German language proficiency without the presence of an accompanying interpreter.

Recruitment was conducted out seven days a week between 10:00 am to 10:00 pm by trained study personnel – including medical students and ED staff released from clinical duties – who completed a one-hour standardized training module. Eligible patients were recruited as a convenience sample by systematically approaching those patients in waiting and treatment areas. After obtaining informed consent, participants completed the digital questionnaire either on a study tablet or their own mobile device. Study teams co-ordinated hand-overs at shift changes and reminded participants not to enrol more than once. Repeated ED attendances by the same individual were documented as separate visits.

### Study outcomes

The primary outcome was the mode of ED presentation (EMS transport vs. self-referred walk-in), stratified by age, presenting complaint, perceived urgency perception and regional setting. Secondary outcomes comprised awareness of the national on-call service number (116117), willingness to engage with digital self-assessment tools and regional variation in EMS utilisation.

### Survey instrument

The questionnaire was designed to assess patient characteristics relevant to emergency care decision-making, including prehospital care-seeking behaviour, attitudes toward alternative care pathways, and openness to digital self-assessment tools. The German-language questionnaire comprised 18 main items (see additional file 1):

- Sociodemographic (adapted from the German Health Interview and Examination Survey for Adults (DEGS)) [8].
- Presenting complaints (classified according to Canadian Emergency Department Information System (CEDIS) [9].
- Perceived urgency (five-level single-choice: not urgent, less urgent, urgent, very urgent, emergency).
- Pain intensity (0–10 numeric rating scale [10]).
- Consultation motives (multiple responses allowed) [11].
- Outpatient care utilisation before ED visit.
- Awareness of the national on-call number 116117.
- Hypothetical willingness to use a digital self-assessment tool.

The questionnaire was piloted in one ED (*n* = 18; varied age, sex, presenting complaint, arrival mode) to assess clarity and comprehensibility. This resulted in the rewording of one item and the addition of another.

### Data management and preparation

Educational level was categorised using the International Standard Classification of Education (ISCED) into low, medium and high [12]. Pain intensity was classified into low (1–3), medium (4–6) and high (7–10). Multiselect responses were preserved. Data were collected anonymously and stored separately from clinical records; no linkage with hospital information systems was performed.

### Regional classification

Regional setting was defined using the European Union´s urban-rural typology based on the Nomenclature of territorial units for statistics [6]: predominantly rural (< 50% of the population residing in urban clusters), intermediate (50–80%) and predominantly urban regions (> 80%) (Table 1). This typology was chosen for its cross-national comparability within the European Union.

### Statistical analysis

Descriptive statistics were used to report frequencies and proportions. Group differences between EMS-transported and walk-in patients were assessed using Mann–Whitney U-test (continuous/ordinal) and χ²-test (categorical). The phi coefficient quantified binary associations. A multivariable logistic regression model was fitted to predict mode of arrival (EMS vs. walk-in) in the full cohort (complete cases *n* = 7,848). Covariates included age; sex; educational attainment; federal state; migration background; perceived urgency; presenting complaints; symptom duration; prior ambulatory contact; seeking online health information and awareness of the national on-call service 116117.

Results are reported as odds ratios (ORs) with 95% confidence intervals (CIs) and two-sided α = 0.05. No centre-level clustering or random effects were applied. Sensitivity analyses were performed. Model goodness-of-fit was assessed using likelihood-ratio tests. ‘No answer’ responses were coded as missing and analyses was performed using complete cases only. In addition, selected outcomes such as readiness to use digital self-assessment were analysed in age-stratified subgroups to assess variation by age. Analyses were performed using IBM SPSS Statistics v29 (IBM Corp, Armonk NY, USA) and R v4.1.2 (R Foundation for Statistical Computing, Vienna, Austria).

### Ethics

The study was conducted in accordance with the Declaration of Helsinki. Ethics approval was granted by Charité – Universitätsmedizin Berlin (EA2/037/24) and endorsed by the Bavarian State Medical Association (mb24034), Ludwig‑Maximilian University (24‑0453) and Technical University of Munich (2024‑230‑S‑CB). Trial registration: DRKS00033986 and DRKS00034961.

### Role of the funding source

The Central Research Institute of Ambulatory Health Care in Germany and the Bavarian Association of Statutory Health Insurance Physicians funded the study. The Central Research Institute of Ambulatory Health Care in Germany contributed to design, data collection, analysis, interpretation and manuscript preparation. The Bavarian Association of Statutory Health Insurance Physicians contributed to data collection and manuscript preparation.

## Results

### Study population

**Fig. 1:**
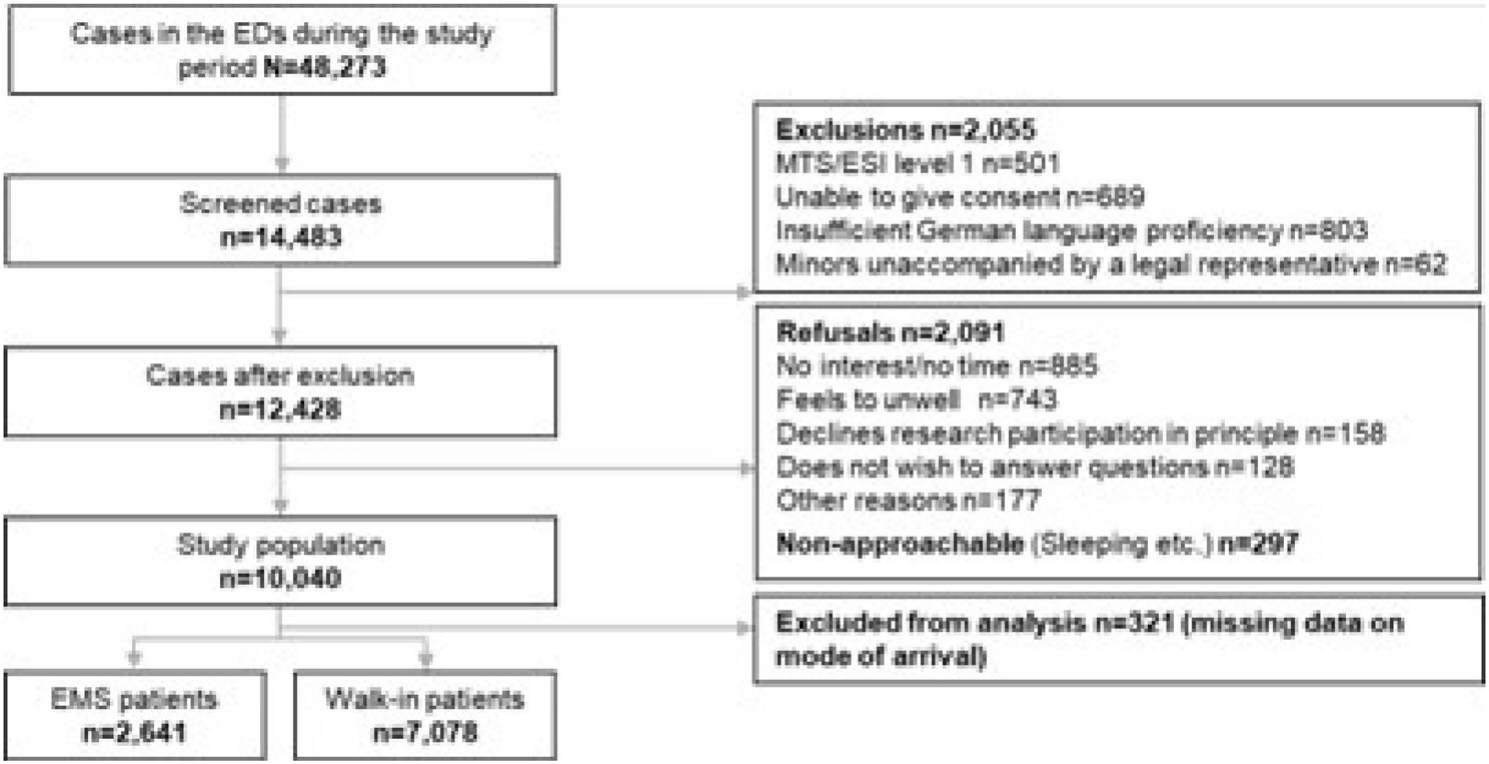
Flow chart of participant inclusion. Legend: 321 patients were excluded due to missing data on mode of arrival. Abbreviations: ED = emergency department; EMS = emergency medical services; MTS = Manchester Triage System; ESI = Emergency Severity Index

Of 10,040 participants, 321 were excluded due to missing data on mode of arrival, yielding a total of 9,719 for the analysis. Overall, 2,641 (27.2%) arrived via EMS and 7,078 (72.8%) self-referred as walk-ins (Fig. 1).

Patients arriving via EMS were older than walk-ins. Nearly half of EMS patients (47.7%) were aged ≥ 70 years, compared with 17.8% of walk-ins. Sex distribution was comparable between groups (*p* = 0.603). Educational profiles differed (*p* < 0.001). First-generation migration background was less frequent in the EMS cohort (13.7% vs. 18.3%; *p* < 0.001) (Table 2).

**Table 2:** Patient characteristics by mode of ED arrival. Data are n (%) unless stated otherwise

Characteristics	All patients (*n* = 9,719)	EMS (*n* = 2,641)	Walk-in (*n* = 7,078)	*p* value
**Age (n = 9,669)**				
< 18 years	658 (6.8%)	55 (2.1%)	603 (8.6%)	< 0.001
18–29 years	1,395 (14.4%)	218 (8.3%)	1,177 (16.7%)	< 0.001
30–39 years	1,284 (13.3%)	196 (7.5%)	1,088 (15.5%)	< 0.001
40–49 years	1,175 (12.2%)	217 (8.3%)	958 (13.6%)	< 0.001
50–59 years	1,316 (13.6%)	302 (11.5%)	1,014 (14.4%)	< 0.001
60–69 years	1,333 (13.8%)	388 (14.8%)	945 (13.4%)	< 0.001
70–79 years	1,210 (12.5%)	480 (18.3%)	730 (10.4%)	< 0.001
≥ 80 years	1,298 (13.4%)	773 (29.4%)	525 (7.5%)	< 0.001
**Sex (n = 9,631)**				
Female	4,772 (49.5%)	1,311 (50.0%)	3,461 (49.4%)	0.603
Male	4,859 (50.5%)	1,312 (50.0%)	3,547 (50.6%)	0.603
**Migration (n = 9,582)**				
None	7,363 (76.8%)	2,102 (80.9%)	5,261 (75.3%)	< 0.001
First-generation	1,632 (17.0%)	355 (13.7%)	1,277 (18.3%)	< 0.001
Second-generation	587 (6.1%)	142 (5.5%)	445 (6.4%)	< 0.001
**Educational attainment (n = 8,813)**				
Low (ISCED 0–2)	1,182 (13.4%)	310 (13.0%)	872 (13.5%)	< 0.001
Medium (ISCED 3–4)	4,499 (51.0%)	1,329 (55.9%)	3,170 (49.2%)	< 0.001
High (ISCED 5–8)	3,132 (35.5%)	737 (31.0%)	2,395 (37.2%)	< 0.001
**Presenting complaint**^†^ **(multi-response; n = 9,720)**				
General/constitutional	640 (6.6%)	230 (8.7%)	410 (5.8%)	< 0.001
Pain	2,736 (28.3%)	600 (22.8%)	2,136 (30.3%)	< 0.001
Injury/trauma	2,463 (25.5%)	658 (25.0%)	1,805 (25.6%)	0.540
Cardio-respiratory	1,593 (16.5%)	685 (26.1%)	908 (12.9%)	< 0.001
Abdominal	1,001 (10.4%)	248 (9.4%)	753 (10.7%)	0.070
Neurological/psychiatric	775 (8.0%)	306 (11.6%)	469 (6.7%)	< 0.001
Dermatologic	238 (2.5%)	23 (0.9%)	215 (3.1%)	< 0.001
Otolaryngology (ENT)	349 (3.6%)	32 (1.2%)	317 (4.5%)	< 0.001
Urological	364 (3.8%)	98 (3.7%)	266 (3.8%)	0.907
Gynecological	159 (1.6%)	11 (0.4%)	148 (2.1%)	< 0.001
Ophthalmic	216 (2.2%)	13 (0.5%)	203 (2.9%)	< 0.001
**Pain Intensity (n = 6,575)**				
Low (1–3)	990 (15.1%)	203 (13.1%)	787 (15.7%)	< 0.001
Medium (4–6)	2,339 (35.6%)	490 (31.6%)	1,849 (36.8%)	< 0.001
High (7–10)	3,246 (49.4%)	858 (55.3%)	2,388 (47.5%)	< 0.001
**Prior contact attempt before ED (n = 9,720)**				
No attempt	5,217 (54.0%)	1,859 (70.7%)	3,358 (47.7%)	< 0.001
General practitioner	2,872 (29.7%)	483 (18.4%)	2,389 (33.9%)	< 0.001
Specialist	1,071 (11.1%)	103 (3.9%)	968 (13.8%)	< 0.001
National on-call service number (116117)	516 (5.3%)	124 (4.7%)	392 (5.6%)	0.097
Out-of-hours clinic	140 (1.4%)	13 (0.5%)	127 (1.8%)	< 0.001

χ² test comparing EMS vs. walk-in for each variable; for multi-response items, p values refer to category-wise 2 × 2 tests. † Multiple responses permitted; percentages use the corresponding arrival-mode denominator and therefore do not sum to 100%. Abbreviations: ED=emergency department; EMS=emergency medical services; ENT = ear, nose and throat; ISCED=International Standard Classification of Education, OR=Odds Ratio

Cardio-respiratory complaints were twice as prevalent among EMS patients compared with walk-ins (26.1% vs. 12.9%; *p* < 0.001), whereas pain was the main symptom among walk-ins (30.3% vs. 22.8%; *p* < 0.001). Pain intensity also differed significantly by mode of arrival (*p* < 0.001): EMS patients reported high pain levels more frequently (55.3% vs. 47.5%; *p* < 0.001), while moderate pain was more common among walk-ins (36.8% vs. 31.6%; *p* < 0.001). Neurological or psychiatric complaints (11.6% vs. 6.7%; *p* < 0.001) and general/constitutional symptoms (8.7% vs. 5.8%; *p* < 0.001) were more common among EMS patients. Low-prevalence complaint categories (dermatologic, otolaryngological, gynaecological and ophthalmic) were concentrated among walk-ins (all *p* < 0.001). EMS patients less frequently attempted general practitioner (GP) contacts before the ED visit (18.4% vs. 33.9%; *p* < 0.001). Prior contact with the national on-call service (116117) was low and did not differ between groups (4.7% vs. 5.6%; *p* = 0.097) (Table 2).

### Consultation motives and readiness for digital self-assessment

Among EMS patients, the predominant reason for ED attendance was a decision made by emergency personnel (53.3%) (Table 3). In contrast, walk-in patients more frequently reported patient-initiated medical motives – such as symptom severity, fear or the desire for a second opinion – as well as external delegation (35.0% cited physician referral; all *p* < 0.001). Convenience-related factors were also more commonly cited by walk-ins. Readiness to use digital self-assessment tools differed significantly (*p* < 0.001): 21.4% of EMS patients and 30.7% of walk-ins expressed willingness; while 74.0% of EMS patients and 62.1% of walk-ins declined its use.

**Table 3:** Consultation motives and readiness to use digital self-assessment tools by mode of arrival

	All patients (*n* = 7.297; multiple answers)	EMS	Walk-in	*p* value
**Consultation motives**				
Fear	1,276 (17.5%)	282 (14.1%)	994 (18.8%)	< 0.001
Severity of symptoms	3,669 (50.3%)	908 (45.5%)	2,761 (52.1%)	< 0.001
GP/specialist has decided	2,221 (30.4%)	365 (18.3%)	1,856 (35.0%)	< 0.001
No family doctor / specialist available	519 (7.1%)	44 (2.2%)	475 (9.0%)	< 0.001
Diagnostics and therapy quickly available	608 (8.3%)	110 (5.5%)	498 (9.4%)	< 0.001
Decision by EMS	1,115 (15.3%)	1,064 (53.3%)	51 (1.0%)	< 0.001
Specialists work at the hospital	1,020 (14.0%)	205 (10.3%)	815 (15.4%)	< 0.001
ED always open	745 (10.2%)	93 (4.7%)	652 (12.3%)	< 0.001
ED easily accessible	410 (5.6%)	53 (2.7%)	357 (6.7%)	< 0.001
Former positive experiences in the ED	341 (4.7%)	46 (2.3%)	296 (5.6%)	< 0.001
Do not know any alternative	466 (6.4%)	63 (3.2%)	403 (7.6%)	< 0.001
Second opinion	44 (0.6%)	3 (0.2%)	41 (0.8%)	0.002
**Ready to use digital self-triage tools**				
Yes	2,630 (28.1%)	549 (21.4%)	2,081 (30.7%)	< 0.001
Maybe	603 (6.5%)	119 (4.6%)	484 (7.2%)	< 0.001
No	6,105 (65.4%)	1,904 (74.0%)	4,201 (62.1%)	< 0.001

Multiple responses allowed; percentages may exceed 100%. Abbreviations: ED=emergency department; EMS=emergency medical services; GP=general practitioner

### Predictors of EMS transport

In the multivariable model (*n* = 7,848; Table 4), age was the dominant demographic predictor. Compared with patients aged ≥ 80 years, the likelihood of arriving by EMS declined steadily in younger groups (e.g. 60–69 years OR 0.31 [95% CI 0.25–0.38]; 18–29 years 0.12 [CI 0.09–0.15]). Sex showed no independent association (OR 1.07 [CI 0.95–1.21]; *p* = 0.26). Educational attainment was inversely related with EMS use: compared with low education, both medium (0.74 [CI 0.61–0.90] and high (0.62 [CI 0.50–0.77]) education were linked to lower odds of EMS arrival. Patients with a migration background were marginally less likely to be transported by EMS than those without (OR 0.86 [CI 0.72–0.98]; *p* = 0.022). Attendance at Berlin EDs had significantly higher odds of EMS use (OR 1.52 [CI 1.32–1.75]; *p* < 0.001).

**Table 4:** Adjusted predictors of EMS transport. Adjusted ORs from multivariable logistic regression (complete cases *n* = 7,848). Data are OR (95% CI)

Variable	Adjusted OR (95% CI)	*p* value
**Age (ref. ≥80 years)**		
< 18 years	0.05 (0.03–0.08)	< 0.001
18–29 years	0.12 (0.09–0.15)	< 0.001
30–39 years	0.13 (0.10–0.16)	< 0.001
40–49 years	0.16 (0.13–0.21)	< 0.001
50–59 years	0.22 (0.17–0.27)	< 0.001
60–69 years	0.31 (0.25–0.38)	< 0.001
70–79 years	0.47 (0.38–0.57)	< 0.001
Sex (ref. female)		
Male	1.07 (0.95–1.21)	0.26
**Educational attainment (ref. low)**		
Medium (ISCED 3–4)	0.74 (0.61–0.90)	0.003
High (ISCED 5–8)	0.62 (0.50–0.77)	< 0.001
**Migration (ref no)**		
Any migration	0.86 (0.72–0.98)	0.022
**Perceived urgency (ref. not urgent)**		
Less urgent	0.78 (0.44–1.43)	0.411
Urgent	0.81 (0.47–1.46)	0.475
Very urgent	1.32 (0.76–2.37)	0.344
Emergency	1.88 (1.07–3.39)	0.031
**Presenting complaint**^*†*^ **(binary indicators)**		
General/constitutional	1.85 (1.46–2.34)	< 0.001
Pain	0.94 (0.81–1.09)	0.423
Injury/trauma	0.82 (0.69–0.97)	0.022
Cardio-respiratory	2.19 (1.85–2.61)	< 0.001
Abdominal	1.39 (1.13–1.72)	0.002
Neurological	2.06 (1.65–2.56)	< 0.001
Dermatologic	0.40 (0.21–0.70)	0.002
Otolaryngology (ENT)	0.38 (0.24–0.57)	< 0.001
Urological	1.12 (0.86–1.53)	0.47
Gynaecological	0.38 (0.15–0.78)	0.017
Eye problems	0.25 (0.13–0.45)	< 0.001
**Symptom duration (ref. since today)**		
Since yesterday	0.33 (0.32–0.45)	< 0.001
Since a few days	0.34 (0.28–0.40)	< 0.001
Since one week	0.31 (0.23–0.42)	< 0.001
1 week but < 1 month	0.39 (0.30–0.49)	< 0.001
1 month	0.38 (0.29–0.50)	< 0.001
**Prior ambulatory contact (ref. no contact)**		
Prior contact attempt	0.38 (0.33-0.43)	< 0.001
**Looked for online advice (ref. no)**		
Yes	0.46 (0.38–0.55)	< 0.001
**Aware of 116117 (ref. no)**		
Yes	0.85 (0.75–0.96)	0.01
State (ref. Bavaria)		
Berlin	1.52 (1.32–1.75)	< 0.001

Model fit: Nagelkerke R² = 0.344; Hosmer-Lemeshow test: χ² = 7.424, df = 8, *p* = 0.492. † Presenting-complaint categories (based on the multi-response question in the questionnaire) were entered as separate binary indicators (present vs. absent) in the model, no single reference category. Abbreviations: CI=confidence interval; EMS=emergency medical services; ENT = ear, nose and throat; ISCED=International Standard Classification of Education; OR=odds ratio

Among clinical predictors, cardio-respiratory (OR 2.19 [CI 1.85–2.61]), neurological (OR 2.06 [CI 1.65–2.56]), abdominal (OR 1.37 [CI 1.13–1.72]) and general/constitutional complaints (OR 1.85 [CI 1.46–2.34]) showed the strongest positive association with EMS use, while ophthalmic, otolaryngology, dermatological, gynaecological and injuries/trauma complaints were inversely associated.

Subjective urgency was associated with EMS transport only at the highest category (emergency: OR 1.88 [95% CI 1.07–3.39]); ‘very urgent’, ‘urgent’, and ‘less urgent’ did not differ from ‘not urgent’. Lack of prior outpatient contact strongly predicted EMS use (OR 0.28 [95% CI 0.33–0.43]). Online information seeking behaviours (OR 0.46 [95% CI 0.38–0.55]) and awareness of the 116117-service number (OR 0.85 [95% CI 0.75–0.96]) were associated with lower odds of EMS use.

### Predictors of EMS presentations to predominantly urban vs. intermediate region EDs

In the regional comparison (*n* = 2,289; Table 5), age, educational attainment, migration background, perceived urgency and awareness of the national medical on-call number (116117) were significantly associated with attending a predominantly urban vs. intermediate ED. Among symptom clusters, injury/trauma-related presentations were more frequent in predominantly urban settings (*p* = 0.003; adj. *p* = 0.058).

**Table 5:** Patient characteristics by regional classification. Data are n (%) unless stated otherwise

Characteristic	All EMS patients (*n* = 2, 289)	Intermediate (*n* = 722)	Predominantly urban (*n* = 1,563)	*p* value (adj. Bonferroni *p* Value)
**Age**				
< 18 years	36 (1.6%)	7 (1.0%)	29 (1.9%)	0.001(0.026)
18–29 years	189 (8.3%)	58 (8.0%)	131 (8.4%)	
30–39 years	170 (7.4%)	36 (5.0%)	134 (8.6%)	
40–49 years	196 (8.6%)	50 (6.9%)	146 (9.3%)	
50–59 years	278 (12.2%)	83 (11.5%)	195 (12.5%)	
60–69 years	355 (15.5%)	117 (16.2%)	238 (15.2%)	
70–79 years	415 (18.2%)	133 (18.4%)	282 (18.0%)	
≥ 80 years	646 (28.3%)	238 (33.0%)	408 (26.1%)	
**Sex**				
Female	1,130 (49.4%)	367 (50.8%)	763 (48.7%)	0.365 (1.000)
Male	1,159 (50.6%)	355 (49.2%)	804 (51.3%)	
*Migration*				
Yes	1,856 (81.1%)	630 (87.3%)	1,226 (78.2%)	< 0.001 (0.002)
**Educational attainment**				
Low (ISCED 0–2)	298 (13.0%)	79 (10.9%)	219 (14.0%)	< 0.001 (< 0.001)
Medium (ISCED 3–4)	1,280 (55.9%)	451 (62.5%)	829 (52.9%)	
High (ISCED 5–8)	711 (31.1%)	192 (26.6%)	519 (33.1%)	
*Perceived urgency*				
Not urgent	24 (1.0%)	10 (1.4%)	14 (0.9%)	< 0.001 (0.012)
Less urgent	153 (6.7%)	59 (8.2%)	94 (6.0%)	
Urgent	688(30.1%)	244 (33.8%)	444 (28.3%)	
Very urgent	816 (35.6%)	253 (35.0%)	563 (35.9%)	
Emergency	608 (26.6%)	156 (21.6%)	452 (28.8%)	
**Presenting complaint**†				
General/constitutional	200 (8.7%)	76 (10.5%)	124 (7.9%)	0.048 (0.864)
Pain	518 (22.6%)	166 (23.0%)	352 (22.5%)	0.820 (1.000)
Injury/trauma	560 (24.5%)	148 (20.5%)	412 (26.3%)	0.003 (0.058)
Cardio-respiratory	618 (27.0%)	209 (28.9%)	409 (26.1%)	0.169 (1.000)
Abdominal	222 (9.7%)	88 (12.2%)	134 (8.6%)	0.008 (0.142)
Neurological/psychiatric	271 (11.8%)	73 (10.1%)	198 (12.6%)	0.095 (1.000)
Dermatologic	20 (0.9%)	8 (1.1%)	12 (0.8%)	0.565 (1.000)
Otolaryngology (ENT)	31 (1.4%)	6 (0.8%)	25 (1.6%)	0.202 (1.000)
Urological	84 (3.7%)	19 (2.6%)	65 (4.1%)	0.094 (1.000)
Gynecological	8 (0.3%)	3 (0.4%)	5 (0.3%)	0.713 (1.000)
Ophthalmic	13 (0.6%)	4 (0.6%)	9 (0.6%)	1.000 (1.000)
**Prior contact attempt before ED**				
Attempt	619 (27.0%)	202 (28.0%)	417 (26.6%)	0.527 (1.000)
**Aware of 116117**				
Yes	1,260 (55.0%)	346 (47.9%)	914 (58.3%)	< 0.001 (< 0.001)

χ² test/Fishers Exact test comparing intermediate and predominantly urban for each variable; Fisher’s exact test was applied when expected cell counts were < 5. For polytomous variables (e.g. age, educational attainment, perceived urgency), the p-value refers to the overall group-wise test and is reported once. For binary variables and individual presenting complaint categories, p-values refer to single 2 × 2 comparisons. † Multiple responses permitted; percentages use the corresponding arrival-mode denominator and therefore do not sum to 100%. Abbreviations: ED=emergency department; EMS=emergency medical services; ENT = ear, nose and throat; ISCED=International Standard Classification of Education

Prior outpatient contact did not differ by ED location (28.0% vs. 26.6%, *p* = 0.527; adj. *p* = 1). However, awareness of the national medical on-call number (116117) was significantly higher among patients in predominantly urban EDs (47.9% vs. 58.3%; *p* < 0.001; adj. *p* < 0.001).

## Discussion

In this large multicentre study of nearly 10,000 ED patients in Germany, we identified substantial differences between EMS-transported patients and those self-presenting as walk-ins.

### Patterns of EMS utilisation and patient behaviour

EMS patients in this study were older and more likely to present with cardio-respiratory, neurological or constitutional complaints, whereas walk-in patients more often reported pain and a broader range of low-acuity conditions. This profile is consistent with prior cohorts in which EMS patients were typically older and more likely to present with cardio-respiratory or neurological conditions [13, 14]. More than half of EMS patients stated that the decision to attend the ED was made by professionals – emergency personnel – rather than by patients. In Germany, calls to the emergency dispatch centre (112) commonly prompt EMS attendance and in a high proportion of cases, transport to ED. However, non-conveyance can occur following clinical assessment and in line with local pathways. Non-conveyance pathways and on-scene decision aids – such as community paramedic models [15] and risk-prediction tools [20] – are being developed but require further evaluation regarding safety and undertriage risk [16–19]. Although EMS patients more frequently reported high pain intensity, pain as a presenting category was not independently associated with EMS use suggesting that symptom onset and type, rather than severity alone, may drive EMS activation. Perceived urgency contributed little except at the top of the scale; only presentations rated “emergency” were associated with higher odds of EMS use. Importantly, the absence of prior contact with outpatient care was the strongest behavioural predictor of EMS use, with more than 70% of EMS patients reporting no such attempt. Conversely, uncertainty about suitable alternatives appeared more relevant among walk-in patients, potentially reflecting a lack of clarity on where else to seek help. Notably, over one third of walk-in patients (35.0%) reported that their ED attendance was prompted by a GP or specialist referral, suggesting that ED demand is not solely driven by patient-initiated decisions but also shaped by referral behaviours in ambulatory care. Perceived urgency has been identified as a key driver of EMS use in prior studies [21]. Searching for online information or awareness of the national on-call number (116117) – which does not provide direct physician contact - were associated with lower EMS utilisation, although the proportion who actually called 116117 before ED attendance did not differ significantly between groups. This suggests that awareness of 116117 may function as a marker of broader health-system literacy rather than a directly actionable mechanism. Unmeasured confounders – including prior experience with telephone triage, waiting times, transport availability and distance to the ED – may additionally influence these associations.

### Regional context: predominantly urban vs. intermediate settings

Regional analyses revealed distinct utilisation patterns between predominantly urban and intermediate located EDs. Predominantly urban EDs were more often used by patients with a migration background and those with lower educational attainment, reflecting known sociodemographic clustering in urban healthcare use. Injury-related presentations were also more frequent in predominantly urban settings, while age and sex showed no independent association with setting. Higher perceived urgency was associated with greater odds of predominantly urban ED attendance, suggesting that case-mix severity contributes to urban demand. No significant differences were observed in prior outpatient contact between regions, indicating that barriers to ambulatory care are not confined to intermediate setting. However, patients aware of the 116117-hotline were more likely to attend predominantly urban EDs, although this association may reflect broader sociodemographic or regional differences rather than a direct effect of 116117 awareness. These findings complement existing registry and claims data on regional EMS variation in Germany [22, 23] and add a patient-level behavioural perspective. Whether the observed regional differences reflect patient-level care-seeking behaviour, structural features of the healthcare landscape – such as the availability of ambulatory care and the concentration of tertiary centres in urban areas – or both, cannot be determined from these data. ED selection in EMS cases is likely driven primarily by the hospital’s emergency care level rather than geographical proximity, which may partly explain observed regional patterns.

### Implications for policy and system integration

Two observations merit further investigation for system design. First, the strong association between lack of prior outpatient contact and EMS use points to a potential role for upstream intervention, although the cross-sectional design does not allow conclusions about whether earlier outpatient contact would have changed the care pathway. Second, awareness of the 116117-hotline and engagement with digital information sources were linked to lower EMS utilisation, although actual prior use of 116117 was low and did not differ between groups, indicating that awareness may reflect broader health-system literacy rather than a directly actionable mechanism. Educational attainment and migration background showed independent associations with EMS use – higher education was linked to lower EMS utilisation and having a migration background to slightly lower EMS utilisation – highlighting the need for nuanced, equity-minded approaches rather than one-size-fits-all campaigns. Educational attainment likely reflects a composite of health literacy, socioeconomic resources and familiarity with the healthcare system rather than a direct causal pathway; the cross-sectional design cannot disentangle these mechanisms.

Germany’s current emergency care system offers two separate entry points (emergency EMS dispatch 112 and 116117 for triage, directing and scheduling in low-acuity situations), which often operate in parallel with limited integration. Our findings are consistent with calls to better align EMS dispatch more closely with structured medical advice systems, drawing on international examples such as UK’s NHS 111 and Denmark’s 1813 service. Evidence suggests that integrated telephone triage may contribute to more appropriate care-seeking [24, 25]. In Germany, the recent cabinet draft of a major reform law (the NotfallG) explicitly envisaged closer 112–116117 integration through joint emergency control systems, providing a policy window for such alignment [26, 27]. The extent to which region-specific strategies could contribute to more appropriate utilisation requires further evaluation. Moreover, many EMS control centres still lack standardised, software-guided first assessment, underscoring the need to scale structured call-taking/dispatch to ensure consistent routing [28, 29]. Given lower digital-tool readiness observed among EMS patients, future implementation should consider equitable reach.

### Strengths and limitations

This study has several strengths. To our knowledge, it represents the largest prospective patient survey of EMS arrivals in German EDs to date. By capturing nearly 10,000 cases across predominantly urban and intermediate sites, the study offers granular insights into care-seeking behaviour, presenting complaints and digital self-assessment readiness. The inclusion of a wide range of hospitals – from inner-city university centres to smaller community sites – enabled the analysis of regional gradients that are often overlooked in single-centre studies. A pre-tested, tablet-based questionnaire administered at the point of care minimised recall bias and ensured consistent data capture. Adherence to STROBE guidelines and prospective trial registration further strengthen methodological transparency. Finally, parallel recruitment of walk-in patients enabled direct comparison with EMS patients across sociodemographic, clinical, behavioural and digital engagement.

Limitations include the cross-sectional design, which precludes causal inference and reliance on self-reported data, which may be prone to misclassification and social desirability bias [30, 31]. Responses may have been influenced by the acute context of illness or injury and the ED environment at the time of survey. Patients with limited German proficiency were underrepresented, potentially underestimating migration-related disparities. The sickest EMS patients (triage red/1) were excluded due to ethical constraints and no linkage to clinical records was made to verify diagnoses or outcomes. The absence of linkage to clinical records or patient outcomes means that we cannot assess whether individual care-seeking decisions were clinically appropriate. The descriptive patterns identified here should therefore not be interpreted as evidence of inappropriate utilisation. Patients younger than 18 years (6.8%, *n* = 658) were included but not analysed separately, as the small number of paediatric EMS cases (*n* = 55) precluded meaningful subgroup analysis. The over-representation of tertiary centres in predominantly urban areas may partly explain observed regional differences. The Eurostat settlement typology [6] does not directly capture healthcare infrastructure variables such as EMS response times, proximity to EDs or availability of primary care alternatives and the binary distinction between predominantly urban and intermediate regions may mask within-category variation. Germany’s physician-staffed ambulance model and the absence of patient co-payments may produce utilisation patterns that differ from paramedic-led or fee-for-service systems elsewhere, which may limit generalisability to other European settings. Finally, missing data handled by complete-case analysis; overall proportion missing data was low, so material bias is unlikely. The large sample size increases the likelihood of detecting statistically significant associations of limited clinical magnitude. Because the survey was conducted anonymously without personal identifiers, repeated attendances by the same individual cannot be quantified, which may introduce minor non-independence.

## Conclusions

Patients arriving by EMS differ systematically from self-presenters in age, case-mix, symptom onset and pre-hospital behaviour. The absence of prior outpatient contact was the strongest behavioural predictor of EMS use and awareness of the 116117 service was associated with lower odds of EMS transport, although actual prior use of 116117 was low and did not differ between groups. These findings highlight opportunities for further research into whether closer integration of structured medical advice services with EMS dispatch, combined with improved availability of ambulatory alternatives, could contribute to more appropriate resource allocation. Whether such approaches can achieve safe and effective patient guidance requires prospective evaluation with clinical outcome linkage.

## Supplementary Information

Below is the link to the electronic supplementary material.

Supplementary Material 1

## Data Availability

The datasets used and analysed during the current study are available from the corresponding author on reasonable request. The full study protocol is also available on request or via the German Clinical Trials Register (DRKS00033986; DRKS00034961).

## Declarations

### Patient and public involvement

No formal patient or public involvement was undertaken in the design, conduct or reporting of this study. The questionnaire was piloted with ED patients to assess clarity and comprehensibility, but this did not constitute structured PPI.

### Research Group

Research Group: Harald Dormann (Department of Emergency Medicine Fürth Hospital), Rene Hartensuer (Centre for Acute and Emergency Medicine Aschaffenburg-Alzenau Hospital), Thomas Händl (Department of Emergency Medicine Garmisch-Partenkirchen Hospital), Manuel Holder (Bavarian Association of Statutory Health Insurance Physicians), Marleen Pfeiffer (Department of Emergency Medicine Wertach Clinics Bobingen and Schwabmünchen), Felix Rockmann (Emergency Centre Barmherzige Brüder Hospital Regensburg), Peter Rupp (Department of Emergency Medicine Starnberg Clinic), Markus Wehler (Department of Emergency Medicine University Hospital Augsburg), Markus Wörnle (Emergency Department at Campus Innenstadt Ludwig Maximilian University of Munich).

## Abbreviations

CEDIS: Canadian emergency department information system
CI: Confidence interval
DEGS: German health interview and examination survey for adults
ED: Emergency department
EMS: Emergency medical service
GP: General practitioner
NHS: National Health Service
OR: Odds ratio
UK: United Kingdom

## References

[1] Morley C, Unwin M, Peterson GM, Stankovich J, Kinsman L. Emergency department crowding: A systematic review of causes, consequences and solutions. Bellolio F, editor. PLoS ONE. 2018;13:e0203316. 10.1371/journal.pone.0203316 30161242 10.1371/journal.pone.0203316 PMC6117060

[2] Greiner F, Erdmann B, Thiemann VS, Baacke M, Grashey R, Habbinga K, et al. Der AKTIN-Monatsbericht: Plädoyer für ein standardisiertes Reporting in der Notaufnahme: Entwicklung und Implementierung eines internen Berichtswesens auf Basis des Datensatzes Notaufnahme. Notf Rettungsmedizin. 2023;26:416–25. 10.1007/s10049-021-00910-z.

[3] Holzinger F, Oslislo S, Resendiz Cantu R, Möckel M, Heintze C. Diverting less urgent utilizers of emergency medical services to primary care: is it feasible? Patient and morbidity characteristics from a cross-sectional multicenter study of self-referring respiratory emergency department consulters. BMC Res Notes. 2021;14:113. 10.1186/s13104-021-05517-8. 33761978 10.1186/s13104-021-05517-8 PMC7992314

[4] Strum RP, Mowbray FI, Worster A, Tavares W, Leyenaar MS, Correia RH, et al. Examining the association between paramedic transport to the emergency department and hospital admission: a population-based cohort study. BMC Emerg Med. 2021;21:117. 10.1186/s12873-021-00507-2. 34641823 10.1186/s12873-021-00507-2 PMC8506085

[5] Holzinger F, Kümpel L, Resendiz Cantu R, Alberter A, Möckel M, Heintze C. Could low-acuity emergency medical services patients be redirected to primary care? Findings from a multi-center survey in Berlin, Germany. BMC Emerg Med. 2025;25:138. 10.1186/s12873-025-01295-9. 40739617 10.1186/s12873-025-01295-9 PMC12312431

[6] European Commission, Statistical Office of the European Union. Methodological manual on territorial typologies: 2018 edition. LU: Publications Office. 2019. 10.2785/930137.

[7] Dewidar O, Shamseer L, Melendez-Torres GJ, Akl EA, Ramke J, Wang X, et al. Improving the Reporting on Health Equity in Observational Research (STROBE-Equity): Extension Checklist and Elaboration. JAMA Netw Open. 2025;8:e2532512. 10.1001/jamanetworkopen.2025.32512. 40899932 10.1001/jamanetworkopen.2025.32512

[8] Luppa M, Giersdorf J, Riedel-Heller S, Prütz F, Rommel A. Frequent attenders in the German healthcare system: determinants of high utilization of primary care services. Results from the cross-sectional German health interview and examination survey for adults (DEGS). BMC Fam Pract. 2020;21:10. 10.1186/s12875-020-1082-9. 31931727 10.1186/s12875-020-1082-9 PMC6958724

[9] Brammen D, Greiner F, Dormann H, Mach C, Wrede C, Ballaschk A, et al. Lessons learned in applying the International Society for Pharmacoeconomics and Outcomes Research methodology to translating Canadian Emergency Department Information System Presenting Complaints List into German. Eur J Emerg Med. 2018;25:295–9. 10.1097/MEJ.0000000000000450. 28145941 10.1097/MEJ.0000000000000450 PMC6039420

[10] Hassett AL, Whibley D, Kratz A, Williams DA. Measures for the Assessment of Pain in Adults. Arthritis Care Res. 2020;72:342–57. 10.1002/acr.24222. 10.1002/acr.24222 33091243

[11] Holzinger F, Oslislo S, Möckel M, Schenk L, Pigorsch M, Heintze C. Self-referred walk-in patients in the emergency department – who and why? Consultation determinants in a multicenter study of respiratory patients in Berlin, Germany. BMC Health Serv Res. 2020;20:848. 10.1186/s12913-020-05689-2. 32912185 10.1186/s12913-020-05689-2 PMC7481545

[12] Schroedter JH, Lechert Y, Lüttinger P. Die Umsetzung der Bildungsskala ISCED-1997 für die Volkszählung 1970, die MikrozensusZusatzerhebung 1971 und die Mikrozensen 1976–2004 (Version 1). Mannheim: Zentrum für Umfragen, Methoden und Analysen; 2006.

[13] Rößler M, Schulte C, Bobeth C, Wende D, Karagiannidis C. Inanspruchnahme des Rettungsdienstes im Kontext von Krankenhausaufnahmen [Internet]. Berlin: BARMER Institut für Gesundheitssystemforschung; 2024. Accessed 14 Jan 2026. https://www.bifg.de/media/dl/ePaper/ePaper_Notfallversorgung_BF.pdf

[14] Christensen EF, Larsen TM, Jensen FB, Bendtsen MD, Hansen PA, Johnsen SP, et al. Diagnosis and mortality in prehospital emergency patients transported to hospital: a population-based and registry-based cohort study. BMJ Open. 2016;6:e011558. 10.1136/bmjopen-2016-011558. 27377636 10.1136/bmjopen-2016-011558 PMC4947831

[15] Sommer A, Rehbock C, Seeger I, Klausen A, Günther U, Schröder H, et al. Zwei Jahre Pilotphase Gemeindenotfallsanitäter in der Region Oldenburg (Niedersachsen): Eine retrospektive Querschnittsstudie zu den Erfahrungen der Mitarbeitenden. Notf Rettungsmedizin. 2025;28:542–51. 10.1007/s10049-022-01079-9.

[16] Dami F, Golay C, Pasquier M, Fuchs V, Carron P-N, Hugli O. Prehospital triage accuracy in a criteria based dispatch centre. BMC Emerg Med. 2015;15:32. 10.1186/s12873-015-0058-x. 26507648 10.1186/s12873-015-0058-x PMC4624668

[17] Kästner A, Lücker P, Fischer L, Laslo T, Henkel B, Ehleben J, et al. The urgent need for patients’ diagnoses and outcome feedback in Germany’s emergency medical services — insights from a web-based survey. BMC Emerg Med. 2025;25:66. 10.1186/s12873-025-01218-8. 40254601 10.1186/s12873-025-01218-8 PMC12010660

[18] Chhabra N, English SW, Butterfield RJ, Zhang N, Hanus AE, Basharath R, et al. Poor prediction of stroke mimics using validated stroke mimic scales in a large academic telestroke network. J Telemed Telecare. 2025;31:1278–84. 10.1177/1357633X241273762. 39158498 10.1177/1357633X241273762

[19] Scala I, Covino M, Rizzo PA, Bisegna M, Marchese D, Bellavia S, et al. A novel stroke mimic prediction score during in-hospital triage for suspected stroke patients: The Stroke Mimics Score (SMS). Eur Stroke J. 2025;23969873251338656. 10.1177/23969873251338654. 10.1177/23969873251338654 PMC12084216 40375550

[20] Miles J, Jacques R, Campbell R, Turner J, Mason S. The Safety INdEx of Prehospital On Scene Triage (SINEPOST) study: The development and validation of a risk prediction model to support ambulance clinical transport decisions on-scene. PLoS ONE. 2022;17:e0276515. 10.1371/journal.pone.0276515. 36383548 10.1371/journal.pone.0276515 PMC9668173

[21] Dahmen J, Brettschneider P, Poloczek S, Pommerenke C, Wollenhaupt L, Breuer F. „Warum wird der Notruf 112 gewählt? – Befragung zum Notrufverhalten der Berliner Bevölkerung. Notf Rettungsmedizin. 2024;27:42–50. 10.1007/s10049-021-00954-1.

[22] Roessler M, Schulte C, Bobeth C, Petrautzki I, Korthauer L, Dahmen J, et al. Regional differences, repeated use, and costs of emergency medical services in Germany. Med Klin - Intensivmed Notfallmedizin. 2024. 10.1007/s00063-024-01189-x 10.1007/s00063-024-01189-x PMC12504377 39320466

[23] Wolff J, Breuer F, Pommerenke C, Dahmen J. Strukturparameter des Rettungswesens in Deutschland – Ergebnisse einer bundesweiten Abfrage und politische Einordnung. Notf Rettungsmedizin. 2024. 10.1007/s10049-024-01408-0.

[24] Egan M, Murar F, Lawrence J, Burd H. Identifying the predictors of avoidable emergency department attendance after contact with the NHS 111 phone service: analysis of 16.6 million calls to 111 in England in 2015–2017. BMJ Open. 2020;10:e032043. 10.1136/bmjopen-2019-032043. 32152158 10.1136/bmjopen-2019-032043 PMC7066618

[25] Zinger ND, Blomberg SN, Lippert F, Krafft T, Christensen HC. Impact of integrating out-of-hours services into Emergency Medical Services Copenhagen: a descriptive study of transformational years. Int J Emerg Med. 2022;15:40. 10.1186/s12245-022-00442-4. 36008756 10.1186/s12245-022-00442-4 PMC9414103

[26] Referentenentwurf des Bundesministeriums für Gesundheit. Entwurf eines Gesetzes zur Reform der Notfallversorgung (NotfallGesetz – NotfallG). Juni 7. 2024. Accessed 14 Jan 2026. https://www.bundesgesundheitsministerium.de/fileadmin/Dateien/3_Downloads/Gesetze_und_Verordnungen/GuV/N/NotfallGesetz_RefE.pdf

[27] Breuer F, Brettschneider P, Poloczek S, Pommerenke C, Wolff J, Dahmen J. Quo vadis, gemeinsames Notfallleitsystem? Standardisierte Notrufabfrage in der Berliner Leitstelle. Notf Rettungsmedizin. 2024;27:640–9. 10.1007/s10049-022-01073-1. 10.1007/s10049-022-01073-1 PMC9449959 36090676

[28] Kraus P, Greiner F, Ebmeyer U, Brammen D. Umsetzung der standardisierten und strukturierten Notrufabfrage in deutschen Rettungsleitstellen im Jahr 2019: Ergebnisse einer bundesweiten Erhebung. Notf Rettungsmedizin. 2022. 10.1007/s10049-022-01099-5.

[29] Gistrichovsky M, Breuer F, Albrecht K, Patjens M, Maurer A, Dax F. Standardisierte Notrufabfrage in der Integrierten Leitstelle: Effiziente Notrufbearbeitung rettet Leben! Notfallmedizin Up2date. 2025;20:161–81. 10.1055/a-2415-4592.

[30] Mann CJ. Observational research methods. Research design II: cohort, cross sectional, and case-control studies. Emerg Med J. 2003;20:54–60. 10.1136/emj.20.1.54. 12533370 10.1136/emj.20.1.54 PMC1726024

[31] Althubaiti A. Information bias in health research: definition, pitfalls, and adjustment methods. J Multidiscip Healthc. 2016;211. 10.2147/JMDH.S104807. 10.2147/JMDH.S104807 PMC4862344 27217764

