# Supplementary material for: Comparing emergency medical service and walk-in patients in German emergency departments: a prospective multicentre survey
Source: BMC Emerg Med. 2026 Apr 14;26:107. doi: 10.1186/s12873-026-01578-9 (PMC13081604; doi:10.1186/s12873-026-01578-9)
Supplement: Supplementary file 1 — Supplementary Material 1 [file 12873_2026_1578_MOESM1_ESM.docx]

| **Q1: Please enter the postal code of your place of residence.** |
| --- |
|  |

| **Q2: For what medical reason did you come to the emergency department today?** | |
| --- | --- |
|  | General complaints:   - 🞏 Fever - 🞏 Nonspecific symptoms / general weakness |
|  | Pain:   - 🞏 Back pain - 🞏 Pain in upper extremities - 🞏 Pain in lower extremities |
|  | Injuries:   - 🞏 Injury upper extremities - 🞏 Injury lower extremities - 🞏 Back/spinal injury - 🞏 Head/face injury - 🞏 Other injury |
|  | Cardiovascular / Respiratory:   - 🞏 Chest pain - 🞏 Irregular heartbeat - 🞏 High blood pressure - 🞏 Other cardiovascular problems - 🞏 Shortness of breath - 🞏 Coughing/mucus - 🞏 Other respiratory complaints |
|  | Abdominal complaints:   - 🞏 Abdominal pain - 🞏 Diarrhea, nausea/vomiting - 🞏 Other gastrointestinal problems |
|  | Neurological / Psychiatric complaints:   - 🞏 Vertigo - 🞏 Headache - 🞏 Weakness in extremities / signs of stroke - 🞏 Other neurological complaints - 🞏 Mental health issues |
|  | Skin:   - 🞏 Rash - 🞏 Other skin problems |
|  | Ear, nose and throat:   - 🞏 Ear problems - 🞏 Nosebleeds or other nasal problems - 🞏 Throat or oral problems |
|  | Urology:   - 🞏 Flank pain - 🞏 Urinary tract complaints - 🞏 Other urological problems |
|  | Genecology:   - 🞏 Pregnancy-related problems - 🞏 Other gynecologically problems |
|  | Other / Rare:   - 🞏 Eye problems - 🞏 Other unlisted complaints |
|  | Other reasons: ___________________________________________________________ |
|  | Don’t know / No answer |

| **Q3: Did you come to the emergency department today because of pain?** | |
| --- | --- |
|  | Yes |
|  | No |
|  | Don’t know / No answer |
| **Q3.1: [If Yes]: How severe was your pain at the time of registration in the emergency department?** | |
| No pain Very severe pain  1 2 3 4 5 6 7 8 9 10 | |

| **Q4: How long have you had the symptoms that made you come to the emergency department today?** | |
| --- | --- |
|  | Since today |
|  | Since yesterday |
|  | For several days |
|  | For one week |
|  | For more than one week, but less than one month |
|  | For more than one month |
|  | Don’t know / No answer |
| **Q4.1: [If answered 2-5] Have your symptoms changed during the 24 hours before you arrived at the emergency dep3artment?** | |
|  | The symptoms have increased acutely |
|  | The symptoms have increased gradually |
|  | The symptoms have remained the same |
|  | The symptoms have subsided |
|  | Don’t know / No answer |

| **Q5: How urgent do you consider your symptoms to be?** | |
| --- | --- |
|  | Emergency – I need to be seen by a doctor immediately |
|  | Very urgent – I need to be seen by a doctor as soon as possible |
|  | Urgent – I need to be seen by a doctor today |
|  | Less urgent – but I am worried / others are worried about me |
|  | Not urgent |
|  | Don’t know / No answer |

| **Q6: Who decided that you should come to the emergency department?** | |
| --- | --- |
|  | I decided myself |
|  | Passersby / strangers |
|  | Relatives, friends, acquaintances, colleagues, or supervisors |
|  | General practitioner / specialist |
|  | Medical on-call service (116117) |
|  | On-call practice at the hospital |
|  | Another hospital referred me |
|  | Nursing home / home care service |
|  | Emergency medical services |
|  | Emergency services control centre (112) |
|  | Don’t know / No answer |

| **Q7^[[1]](#footnote-2)^: How did you get to the emergency department today?** | |
| --- | --- |
|  | Independently or with private support (on foot, by public transport, car, or taxi) |
|  | By emergency medical services patient transport services |
|  | Don’t know / No answer |
| **Q7.1: [If arrived by emergency medical services or patient transport services] Who called the emergency medical services or patient transport services?** | |
|  | Myself |
|  | Passersby / strangers |
|  | Relatives, friends, acquaintances, colleagues, or supervisors |
|  | General practitioner / specialist |
|  | Medical on-call service (116117) |
|  | Another hospital referred me |
|  | Nursing home / home care service |
|  | Don’t know / No answer |

| **Q8: Before coming to the emergency department, did you try to contact a doctor’s office, the medical on-call service (116117), or a on-call practice?** *(Multiple answers possible)* | |
| --- | --- |
|  | Yes, general practitioner |
|  | Yes, specialist |
|  | Yes, medical on-call service (116117) |
|  | Yes, on-call practices |
|  | No |
|  | Don’t know / No answer |
| **Q8.1 [If yes] Were you able to reach someone?** | |
|  | Yes |
|  | No |
|  | Don’t know / No answer |
| **Q8.1.1 [If yes] Please provide more details.** | |
|  | General practitioner / Specialist:   - 🞏 I had contact with the doctor. I was told to go directly to the emergency department   - 🞏 I received a referral   - 🞏 I received an admission   - 🞏 I did not receive any documents - 🞏 I had no direct contact with the doctor, but I was told to go to the emergency department |
|  | Medical on-call service (116117):   - 🞏 The staff referred me to the emergency department - 🞏 The recommendation was not suitable for me - 🞏 The telephone contact alone was nonsatisfying |
|  | On-call practices:   - 🞏 A medical assistant or nurse referred me to the emergency department - 🞏 The doctor referred me to the emergency department |
|  | Don’t know / No answer |
| **Q8.1.2 [If no] Please provide more details.** | |
|  | General practitioner / Specialist:   - 🞏 I could not reach anyone:   - 🞏 Practice was closed   - 🞏 Phone line was busy   - 🞏 Phone waiting time was too long - 🞏 No timely appointment was available:   - 🞏 The appointment would have been tomorrow   - 🞏 The appointment would have been in 2 days   - 🞏 The appointment would have been in 3-7 days   - 🞏 The appointment would have been after 7 days |
|  | Medical on-call service (116117):   - 🞏 I could not reach anyone:   - 🞏 Phone line was busy   - 🞏 Phone waiting time was too long |
|  | Other reasons: __________________________________________________________________ |
|  | Don’t know / No answer |

| **Q9: Why did you decide to come to the emergency department with your symptoms? Because…**  *(Multiple answers possible)* | |
| --- | --- |
|  | … I was afraid |
|  | … my symptoms were very severe |
|  | … I couldn’t reach a general practitioner or specialist (e.g., practice was closed, no appointment available, etc.) |
|  | … my general practitioner or specialist advised me to come |
|  | … the emergency medical services staff advised me to come |
|  | … diagnostic tests or results are quickly available at the hospital |
|  | … there are medical specialists at the hospital |
|  | … the emergency department is always open |
|  | … the emergency department is easy to reach |
|  | … I have had good experiences with this emergency department before |
|  | … I didn’t know where else to go |
|  | … I was looking for a second opinion regarding treatment from my general practitioner or specialist |
|  | Other reasons: ___________________________________________________________________ |
|  | Don’t know / No answer |

| **Q10: In your opinion, could your problem also have been treated by a general practitioner or specialist?** | |
| --- | --- |
|  | Yes |
|  | No |
|  | Don’t know / No answer |

| **Q11: Do you know the number 116117 (medical on-call service)?** | |
| --- | --- |
|  | Yes |
|  | No |
|  | Don’t know / No answer |
| **Q11.1: [If yes] What kind of help or services do you know 116117 offers?** | |
|  | Home visit service |
|  | Appointment scheduling for general practitioners, specialists, and psychotherapists |
|  | Telephone consultation for medical complaints |
|  | I know the number but not the services it offers |
|  | Don’t know / No answer |

| **Q12: Have you looked up information online about your symptoms? (website / app)** | |
| --- | --- |
|  | Yes |
|  | No |
|  | Don’t know / No answer |
| **Q12.1: [If yes] Did you receive a recommendation to go to the emergency department?** | |
|  | Yes |
|  | No |
|  | Don’t know / No answer |

| **Q13: What is your gender?** | |
| --- | --- |
|  | Female |
|  | Male |
|  | Diverse |
|  | Don’t know / No answer |

| **Q14: How old are you?**  *Please assign your age to the appropriate age group.* | |
| --- | --- |
|  | < 18 years |
|  | 18-29 years |
|  | 30-39 years |
|  | 40-49 years |
|  | 50-59 years |
|  | 60-69 years |
|  | - 1. years |
|  | > 80 years |
|  | Don’t know / No answer |

| **Q15: What is the highest level of general education you have completed?** | |
| --- | --- |
|  | Pupil, attending a full-time general education school |
|  | I left school without a graduation certificate |
|  | Basic Certificate of Secondary Education, General Certificate of Secondary Education or Polytechnic Degree |
|  | A level / Entrance requirement for higher education |
|  | Other school graduation |
|  | Don’t know / No answer |

| **Q16: What is your highest vocational training or higher education qualification?** | |
| --- | --- |
|  | No professional qualification |
|  | Apprenticeship or educational training |
|  | Completed training at a technical school, master school, technical college, academy of cooperative education or academy of advanced vocational studies |
|  | Degree from a university, college, university of applied sciences or engineering school |
|  | Still in apprenticeship (including pre-vocational training, internship, etc.) |
|  | Still studying (including internship during studies, compulsory internship) |
|  | Other educational qualification |
|  | Don’t know / No answer |

| **Q17**: **Were you or your parents born in Germany?** | |
| --- | --- |
|  | Yes, I born in Germany |
|  | Yes, I born in Germany, but not my parents |
|  | No |
|  | Don’t know / No answer |

| **Q18: Would you use a digital application to assess your symptoms online?** | |
| --- | --- |
|  | Yes |
|  | No |
|  | Don’t know |
| **Q18.1: [If Yes/Don’t know] Would you follow the recommendation of an online self-assessment if it suggested seeing a practitioner and offered you an immediate appointment?** | |
|  | Yes |
|  | No |
|  | Don’t know / No answer |

| **Is there anything else you would like to share with us?**  *You may use this space for questions or comments.* |
| --- |
|  |

1. Note: In the German healthcare system, emergency medical services (Rettungsdienst, dispatched via 112) and patient transport services (Krankentransport**, i.e. scheduled, non-emergency ambulance transfers**) are distinct services. The study's primary outcome variable refers to emergency medical services only. [↑](#footnote-ref-2)
